# Orbital Lymphoma Masquerading as Orbital Cellulitis

**DOI:** 10.1155/2021/8832783

**Published:** 2021-09-09

**Authors:** B. D. Chaurasiya, Ganesh Agrawal, Santosh Chaudhary, Sangeeta Shah, Anju Pradhan, Poonam Lavaju

**Affiliations:** ^1^Department of Ophthalmology, Narayani Hospital, Birgunj, Nepal; ^2^Department of Ophthalmology, Mechi Eye Hospital, Jhapa, Nepal; ^3^Department of Ophthalmology, B.P. Koirala Institute of Health Sciences, Dharan, Nepal; ^4^Department of Pathology, B.P. Koirala Institute of Health Sciences, Dharan, Nepal

## Abstract

**Background:**

Orbital lymphomas are primarily non-Hodgkin type and can originate from the eyelids, extraocular muscles, soft tissue orbital adnexa, conjunctiva, or lacrimal glands. Orbital malignancies often represent a diagnostic dilemma for clinicians given their varying and atypical presentations.

**Objective:**

To report a case of orbital lymphoma mimicking orbital cellulitis.

**Case:**

A 66-year-old male patient presented with sudden onset of painful proptosis with visual impairment in the left eye for 15 days. On ocular examination, best-corrected visual acuity was 6/12 in the right eye and 2/60 in the left eye, abaxial proptosis with hypertropia, swollen and erythematous eyelids, restricted extraocular movement in all cardinal position of gaze, conjunctival congestion with chemosis and tortuous vessels, sluggish pupillary reaction, and chorioretinal folds in the inferior quadrants. The case was diagnosed as left eye orbital cellulitis, and the patient was treated with broad-spectrum intravenous antibiotics and oral steroids. No clinically discernible response was noted despite 7 days of antibiotics and steroids. Contrast-enhanced computed tomography (CECT) orbit showed features suggestive of orbital lymphoma involving the ipsilateral maxillary and ethmoid sinuses. ENT consultation with diagnostic nasal endoscopy and biopsy was done. Histopathological reports showed features of non-Hodgkin lymphoma.

**Conclusion:**

Orbital malignancies masquerading as orbital cellulitis can pose a diagnostic dilemma. A multidisciplinary approach involving ENT consultation, radiological investigation, and pathological sampling can help achieve a timely diagnosis and appropriate management.

## 1. Introduction

Orbital lymphomas are a common type of non-Hodgkin Lymphoma (NHL). It primarily originates from the eyelids, extraocular muscles, soft tissue orbital adnexa, conjunctiva, or lacrimal glands. It is most commonly located on the extraconal site, mostly anterior in the orbit or beneath the conjunctiva with a typical “salmon-patch appearance.” Lymphoma originating from one of these locations is called primary orbital lymphoma. Lymphoma originating from extraorbital sites with metastatic spread to the orbit is secondary orbital lymphoma [1]. Among all orbital malignancies, 55% are lymphoma [2]. The incidence of orbital lymphoma lies between 1 and 10% of all NHL and 5 and 15% of all extranodal NHL [3, 4]. Orbital lymphoma can be seen from 15 to 70 years, but most cases occur between 50 and 70 years. It is most commonly seen in Asian and European populations with no gender predominance [1]. Of all orbital lymphoma, 75% are unilateral, 25% are bilateral, and 40% are associated with systemic disease [4].

The major factor contributing to the pathophysiology of orbital lymphoma is immune-compromised conditions like ageing, HIV/AIDS, or immunosuppressive drugs. Recently, studies have suggested that pathogens like Chlamydia psittaci, H. pylori, and some viruses are associated with orbital lymphoma [1]. Lymphomas of B-cell lineage are more likely to be associated with symptoms related to the eyes and have extended to the orbit than lymphomas of T-cell or NK-cell lineage [5]. Orbital lymphomas most commonly present with proptosis, a slow-growing palpable mass, or a painless swelling of the eyelids [6]. Orbital cellulitis is an infectious condition of orbit that can lead to blindness or death. Important causes include trauma, upper respiratory tract infection, and sinus infections [7]. This case report is aimed at discussing orbital cellulitis as a rare mode of presentation of orbital lymphoma.

## 2. Case Description

**Figure 1:**
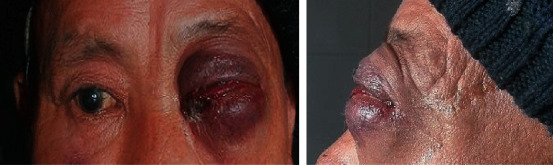
Orbital cellulitis at presentation in the left eye (front and lateral view).

**Figure 2:**
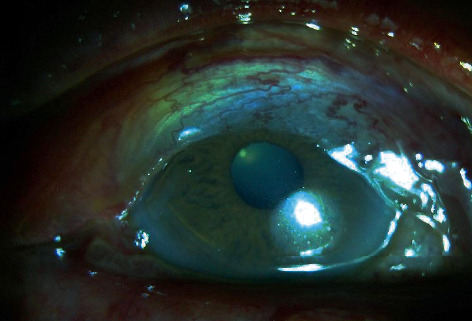
Conjunctival congestion with tortuous vessels in the left eye.

**Figure 3:**
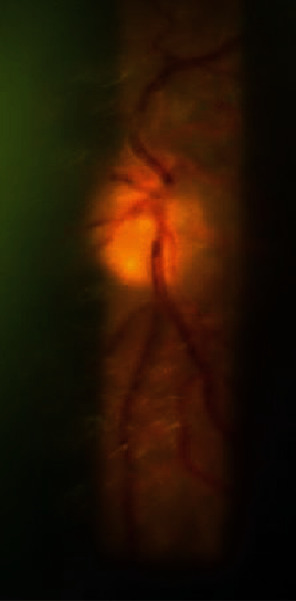
Blurred disc margin with chorioretinal folds in the left eye.

A 66-year-old Nepalese male patient presented in the Outpatient Department (OPD) of Ophthalmology, B.P. Koirala Institute of Health Sciences, with sudden onset of painful proptosis associated with visual impairment in the left eye for 15 days (Figure 1). Other associated features were nasal stuffiness and nasal bleeding on and off but not associated with fever, recent weight loss, or night sweats. On general examination, lymph nodes were not palpable. On examination of the right eye (RE), best-corrected visual acuity (BCVA) was 6/12 with an immature senile cataract of grade I and the rest of ocular examination was within normal limits. On examination of the left eye (LE), BCVA was 2/60 with accurate projection of rays. The left eye showed abaxial proptosis with hypertropia, diffuse swelling, and erythema over the upper and lower eyelids with restricted extraocular movements in all cardinal positions of gaze. There was diffuse conjunctival congestion with chemosis and dilated tortuous vessels over the upper bulbar conjunctiva (Figure 2). Sluggish pupillary reaction in the left eye, lens, and vitreous findings was within a normal limit. A dilated funduscopic examination of the left eye revealed optic disc edema of one diopter elevation with chorioretinal folds in the inferior quadrants (Figure 3). Hertel's exophthalmometry measurement (at base of 112) was 16 mm in the right eye and 28 mm in the left eye. The intraocular pressure was 12 mmHg in the right eye and 30 mmHg in the left.

**Figure 4:**
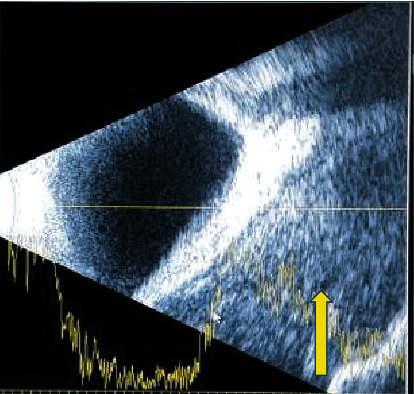
USG B-scan: homogenous mass distorting the globe inferiorly (as shown by an arrow) in the left eye.

USG B-scan of the left eye showed normal intraocular structures with the presence of homogeneous mass behind the globe inferiorly (Figure 4). The patient was admitted with the provisional diagnosis of left eye orbital cellulitis in the stage of an orbital abscess with compressive optic neuropathy. Routine blood investigations were within normal limits. The patient was immediately started on a treatment of broad-spectrum intravenous Ceftriaxone 1 gm BD for 7 days, intravenous Gentamycin 80 mg TDS for 7 days, oral Metronidazole 400 mg TDS for 7 days, eye drop Ofloxacin (0.3%) QID in the left eye, and oral Prednisolone 60 mg OD the next day.

**Figure 5:**
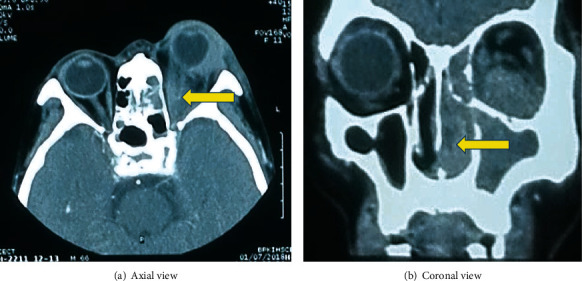
CECT-orbit: contrast enhanced intraorbital mass involving the optic nerve (as shown by an arrow in (a)) and the mass extending to sinonasal cavity (as shown by an arrow in (b)).

**Figure 6:**
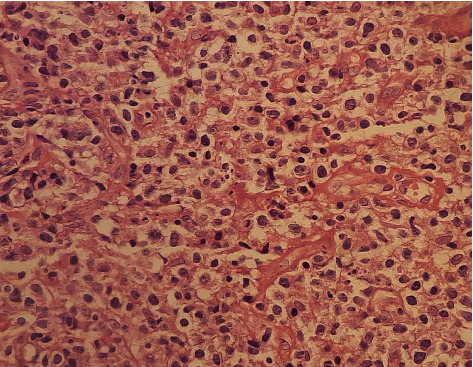
Endoscopic biopsy specimen (H&E x20). Microphotograph shows the H&E-stained section showing diffuse proliferation of atypical cells with apoptotic bodies.

There was no improvement despite two days of intravenous antibiotics and oral steroid; contrast-enhanced computed tomography (CECT) orbit showed homogeneously enhancing soft tissue component in the retroorbital space involving the intraconal compartment including the optic nerve with an adjacent extraconal spread. It also involved the soft tissue of the anterior orbit with homogenous opacity in the ethmoidal and maxillary sinuses with few probable sites of bony erosions (Figures 5(a) and 5(b)). Otorhinolaryngology (ENT) consultation was done, and the patient underwent a diagnostic nasal endoscopic biopsy of the sinus mass. Histopathology examination revealed diffuse proliferation of atypical cells with high nucleocytoplasmic ratio, round to oval nuclei with irregular nuclear membrane, vesicular to coarse chromatin, prominent nucleoli, and scant to moderate amount of cytoplasm, some showing vacuolization. A fair number of mitosis, including atypical ones and apoptotic bodies, were present. Proliferating capillaries and few inflammatory cells were seen in between suggestive of non-Hodgkin Lymphoma (Figure 6). As there was no oncology facility in our institute, the patient was referred to an oncology center for chemotherapy and radiation. On further inquiry with family members, the patient did not receive any further treatment due to unfavorable circumstances. The patient's condition subsequently deteriorated and resulted in death approximately two months after the initial diagnosis.

## 3. Discussion

Non-Hodgkin lymphoma (NHL) is the most common malignant tumor of the orbit [8]. Moslehi et al. reported an increase in the number of systemic and ocular NHL cases over the last 4 decades, which may largely be due to advances in medical diagnostic testing and changes in the classification of lymphomas. In addition, NHL has been linked to environmental factors, such as pesticides, wood preservatives, and solvent exposure; immune disorders, such as occurs with patients on immunosuppressive drugs or those with a transforming virus such as Human Immunodeficiency virus or Epstein Barr virus; patients with connective tissue disorders, such as Sjogren's syndrome, lupus, or rheumatoid arthritis; and infectious diseases, such as chlamydia or hepatitis C [2, 9, 10].

Orbital lymphoma commonly presents with proptosis or palpable mass causing eyelid swelling [6, 11, 12]. It may also be associated with other symptoms like extraocular movement restrictions, diplopia, and eye pain. Visual acuity is decreased mainly due to either optic nerve infiltration or compression caused by mass effect [13].

Lymphoma of orbit can masquerade as orbital cellulitis [14–17]. The pathophysiology behind orbital lymphoma presenting as orbital cellulitis can be due to direct tumor invasion responsible for inflammation of orbital soft tissues [17]. Orbital cellulitis is an infectious condition of the orbital soft tissues posterior to the orbital septum. It mainly occurs due to the direct extension of a paranasal sinus infection which can result in blindness or death [7]. Hence, an investigation suggestive for infection (complete blood count, blood culture, etc.) and immediate treatment should be initiated. In our case, the patient was initially diagnosed with orbital cellulitis based on clinical assessment and he was treated with intravenous antibiotics. However, the persistence of proptosis, conjunctival congestion, eyelid edema, and gradual deterioration of vision even after intravenous antibiotics and oral steroid initiation raised suspicion for an alternative diagnosis such as a malignancy.

Radiological imaging, including a CT scan or MRI, can help diagnose and delineate the extension of the malignant lesion such as orbital lymphoma. Diagnostic confirmation mainly depends on the pathological biopsy for grading and staging of malignancy. Further categorization of orbital lymphoma is aided by immunohistochemistry, making it easy to formulate an appropriate treatment plan. In our case, the orbit seems to be the primary locus of the lesion rather than secondary metastasis, as the normal peripheral blood smear finding makes the chance of hematogenous origin less likely. A more systematic evaluation is still needed, however, to rule out metastasis. Regarding the treatment of localized orbital lymphoma, radiotherapy is appropriate for local control [18, 19]. For metastatic or systemic spread, chemotherapy or a combination of chemotherapy and radiotherapy is highly effective [20]. The prognosis of orbital lymphoma depends upon the various subtypes of lymphoma, as MALT lymphoma has a more favorable outcome than patients with other types of lymphoma [21]. To the best of our knowledge, orbital lymphoma infrequently mimics orbital cellulitis. Hence, to improve the overall prognosis of the conditions as described above, a holistic approach with early diagnosis and management is crucial.

## 4. Conclusion

Orbital lymphoma masquerading as orbital cellulitis can pose a diagnostic dilemma. Orbital imaging like CT scan or MRI should always be performed in a patient with painful proptosis, especially in unilateral cases, to exclude malignancy. A tissue biopsy is needed to confirm the diagnosis of lymphoma. Therefore, a multidisciplinary approach with ENT consultation, radiological investigation, and adequate pathological sampling is necessary to obtain a correct and timely diagnosis to decrease overall morbidity and mortality.

## Data Availability

No data were used to support this study.
